# Dataset of COVID-19 outbreak and potential predictive features in the USA

**DOI:** 10.1016/j.dib.2021.107360

**Published:** 2021-09-10

**Authors:** Arezoo Haratian, Hadi Fazelinia, Zeinab Maleki, Pouria Ramazi, Hao Wang, Mark A. Lewis, Russell Greiner, David Wishart

**Affiliations:** a Department of Electrical and Computer Engineering, Isfahan University of Technology, Isfahan 84156-83111, Iran; b Department of Mathematics and Statistics, Brock University, St. Catharines, ON L2S 3A1, Canada; c Department of Mathematical and Statistical Sciences, University of Alberta, Edmonton, AB T6G 2G1 Canada; d Department of Biological Sciences, University of Alberta, Edmonton, AB T6G 2E9 Canada; e Department of Computing Science, University of Alberta, Edmonton, AB T6G 2E8 Canada; f Alberta Machine Intelligence Institute, Edmonton, AB T5J 3B1 Canada

**Keywords:** COVID-19, Epidemiology, Predictive features, Machine learning

## Abstract

This dataset provides information related to the outbreak of COVID-19 disease in the United States, including data from each of 3142 US counties from the beginning of the outbreak (January 2020) until June 2021. This data is collected from many public online databases and includes the daily number of COVID-19 confirmed cases and deaths, as well as 46 features that may be relevant to the pandemic dynamics: demographic, geographic, climatic, traffic, public-health, social-distancing-policy adherence, and political characteristics of each county. We anticipate many researchers will use this dataset to train models that can predict the spread of COVID-19 and to identify the key driving factors.

## Specifications Table

**Table:** 

Subject	Epidemiology
Specific subject area	COVID-19 outbreak
Type of data	Table
How data were acquired	Collection of publicly available data across literature and databases
Data format	Raw
Parameters for data collection	Data were collected for all US counties since the first COVID-19 case was identified in the country (22 January 2020), until June 10, 2021.
Description of data collection	The data is collected from publically available, online databases. Both the raw dataset, including “missing” values, and the imputed dataset without any missing values, are available to the user.
Data source location	Country: United States of America Data sources for each of the variables included in the data are mentioned in Section 2.
Data accessibility	Repository name: USA covid-19 data Data identification number: 10.6084/m9.figshare.12986069 Direct URL to data: 10.6084/m9.figshare.12986069.v1 Data collection code: 10.5281/zenodo.5231713

## Value of the Data

• We anticipate this dataset will be useful for understanding, modeling, and predicting the COVID-19 pandemic dynamics in the United States with the county-specific spatial-resolution.
• Researchers and governments can benefit from this dataset to gain a better understanding of the COVID-19 pandemic dynamics and inform preventive policies.
• The dataset can provide insights into the wide variety of potential factors affecting the spread of COVID-19.
• We anticipate the wide range of daily features recorded over many months for the large number of counties will be sufficient to estimate the parameter values of mechanistic models, such as the Susceptible-Infected-Recovered (SIR) type models [1], as well as effective machine-learning models [2].

## 1 Data Description

The widespread health, social and economic impact of the current international COVID-19 epidemic makes it crucial to understand pandemic dynamics and improve preventive policies. An effective way to prevent the progression of the outbreak in the affected regions is to identify and if possible, control the factors influencing the spread of the disease in each region. However, the many factors that play a role in the spread of COVID-19 make it challenging to forecast, and hence plan for, the disease spread. Therefore, to examine the impact of potentially influential factors on the disease spread in the United States, we have collected a dataset containing, in each county and for each day since the beginning of the outbreak, the number of confirmed COVID-19 cases and deaths as well as 46 factors that may be relevant to the pandemic dynamics. In addition to the raw dataset, we have prepared a processed version of the dataset, where the missing values are imputed and the abnormal values, e.g., negative counting values, are fixed.

## 2 Experimental Design, Materials and Methods

We rely on authoritative government and academic sources to collect the data, here is to provide the data for each of the features at the county level. For each of the 3142 counties in the US, for each day from the beginning of the disease outbreak in the country, January 22, 2020, until June 10, 2021, the dataset provides the number of confirmed COVID-19 cases and deaths as well as 46 other demographic, geographic, climatic, epidemiological and sociological features that potentially influence the spread and effects of the disease. These features include both fixed and temporal characteristics. Fixed (time-invariant) features generally represent a county's geographic, demographic, and public health information. Temporal (time-varying) features consist of climate factors‏, adherence to social-distancing policies, facility utilization reports, percentage of vaccinated residents, virus pressure from neighboring counties, and a number of tests performed in each state (Table 1). The collection and pre-processing of each of the variables are described below.

**Table 1:** Description of the features.

#	Variable Name	Description	Percentage of values available in the dataset	Type	Finest spatial scale	Date of access to the data source
Target variables						

(1)	COVID-19 confirmed cases	Number of daily COVID-19 confirmed cases	100%	real	county	Jun 10, 2021
(2)	COVID-19 deaths	Number of daily COVID-19 deaths	100%	real	county	Jun 10, 2021

Fixed features						

(3)	Total population	Total population	100%	real	county	Apr 17, 2020
(4)	Population density	Population per square mile	100%	real	county	-
(5)	Proportion female	Total number of females divided by the total population	100%	real	county	-
(6)	Age distribution	Percentage of residents in the age groups: 0-4, 5-9, 10-14, 15-19, 20-24, 25-29, 30-34, 35-39, 40-44, 45-49, 50-54, 55-59, 60-64, 65-69, 70-74, 75-79, 80-84, 85 and older	100%	real vector (18 values, that add up to 1)	county	Apr 17, 2020
(7)	Education level distribution	Percentage of residents with different levels of education: 'less than high school diploma', 'high school diploma', 'some college or associate's degree'	100%	real vector (4 values, that sum to 1)	county	Aug 18, 2020
(8)	Median household income	-	100%	real	county	May 4,2020
(9)	GDP per capita	Gross Domestic Product per capita (economic output divided by the population)	100%	real	county	Apr 27,2020
(10)	Area	Area in square miles	100%	real	county	May 6,2020
(11)	Latitude	Latitude of the county barycenter	100%	real	county	May 1,2020
(12)	Longitude	Longitude of the county barycenter	100%	real	county	May 1,2020
(13)	Housing density	Number of housing units per square mile (Including houses, apartments/flats, mobile homes, and other housing units)	100%	real	county	Apr 17,2020
(14)	Academic population ratio	Total number of residents who are currently university and college students or staff, divided by the total population	100%	real	county	May 4,2020
(15)	Immigrant students ratio	Total number of students who study in this county but are residents of the other states, divided by the total county population	100%	real	county	Sep 10,2020
(16)	Hospital bed ratio	Number of Hospital beds divided by the total population	100%	real	county	May 11,2020
(17)	Intensive care unit (ICU) bed ratio	Number of ICU beds divided by the total population	98%	real	county	May 11,2020
(18)	Ventilator capacity ratio	Number of ventilators divided by the total population	98%	real	county	May 11,2020
(19)	Percent of smokers	Percentage of adult smokers	100%	real	county	May 11,2020
(20)	Percent of diabetes	Percentage of diabetic adults	100%	real	county	May 11,2020
(21)	Religious congregation ratio	Number of active members of Religious congregations divided by the total population	99%	real	county	Apr 17,2020
(22)	Number of meat plants	Number of meat processing plants	100%	discrete	county	Aug 20,2020
(23)	Airport distance	Distance to the nearest international airport with average daily passenger load more than ten	100%	real	county	May 1,2020
(24)	Passenger load ratio	Average daily passenger load of that nearest international airport divided by the total population	100%	real	county	May 20,2020
(25)	Percent of insured residents	Percentage of health insured residents	99%	real	county	May 11,2020
(26)	Death ratio	Number of deaths divided by the total population	97%	real	county	June 21,2020
(27)	Political party	The political party of the governor of each state (0 for Republican and 1 for Democratic)	100%	discrete	state	Apr 17, 2020
(28)	Population ratio in state	Total population of the county, divided by its state population	100%	real	county	-

Temporal features						

(29)	Precipitation	Daily precipitation	73%	real	county	June 10, 2021
(30)	Temperature	Daily average temperature	59%	real	county	June 10, 2021
(31)	Daily state test	Number of total COVID-19 tests performed at each day in the state of the county (including antibody, antigen, and PCR tests)	91%	integer	state	June 10, 2021
(32)	Percent of vaccinated residents	Percent of residents who are fully vaccinated (have second dose of a two-dose vaccine or one dose of a single-dose vaccine)	99%	integer	county	June 10, 2021
(33)	Weekly admission	Weekly average number of adult or pediatric patients who were admitted to an inpatient bed in the county who had confirmed COVID-19 at the time of admission	31%	real	county	June 10, 2021
(34)	weekly reported total ICU beds	Weekly average number of total number of staffed inpatient ICU beds reported by the hospitals in the county	46%	real	county	June 10, 2021
(35)	weekly occupied ICU beds	Weekly average number of total number of staffed inpatient ICU beds that are occupied, reported by the hospitals in the county	45%	real	county	June 10, 2021
(36)	weekly reported total inpatient beds	Weekly average number of total number of staffed inpatient beds (including ICU beds) reported by the hospitals in the county	46%	real	county	June 10, 2021
(37)	weekly occupied inpatient beds	Weekly average number of total number of staffed inpatient beds that are occupied, reported by the hospitals in the county	46%	real	county	June 10, 2021
(38)	Social distancing travel distance grade	Percent change in average distance traveled compared to pre-COVID-19-period (range from A to F) A: >70% decrease B: 55-70% decrease C: 40-55% decrease D: 25-40% decrease F: <25% decrease or increase	99%	nominal	county	June 10, 2021
(39)	Social distancing visitation grade	Percent change in non-essential visitation compared to pre-COVID-19 period (range from A to F) A: >70% decrease B: 65-70% decrease C: 60-65% decrease D: 55-60% decrease F: <55% decrease or increase	82%	nominal	county	June 10, 2021
(40)	Social distancing encounters grade	Percent change in human encounters compared to pre-COVID-19 period (range from A to F) A: >94% decrease B: 82%-94% decrease C: 74%-82% decrease D: 40%-74% decrease F: <40% decrease or increase	99%	nominal	county	June 10, 2021
(41)	Social distancing total grade	Average numerical score of the previous three social distancing factors	99%	nominal	county	June 10, 2021
(42)	Retail and recreation mobility percent change	Percent change in mobility trends in retail shops and recreation centers (including places like restaurants, shopping centers, museums, and libraries) compared to pre-COVID-19 period	49%	real	county	June 10, 2021
(43)	Grocery and pharmacy mobility percent change	Percent change in mobility trends in grocery stores and pharmacies (including places like grocery markets, food warehouses, farmers markets, specialty food shops, drug stores, and pharmacies) compared to pre-COVID-19 period	44%	real	county	June 10, 2021
(44)	Parks mobility percent change	Percent change in mobility trends in parks (including local and national parks, public beaches, marinas, dog parks, plazas, and public gardens) compared to pre-COVID-19 period	18%	real	county	June 10, 2021
(45)	Transit stations mobility percent change	Percent change in mobility trends in transit stations (representing public transport hubs like taxi stands, bus, train, and subway stations) compared to pre-COVID-19 period	28%	real	county	June 10, 2021
(46)	Workplaces mobility percent change	Percent change in mobility trends in places of work compared to pre-COVID-19 period	74%	real	county	June 10, 2021
(47)	Residential mobility percent change	Percent change in mobility trends in places of residence compared to pre-COVID-19 period	42%	real	county	June 10, 2021
(48)	Virus pressure	A measure for virus transmission from neighboring counties, defined as the weighted average of the number of confirmed cases in the adjacent counties (ie, that share a border with this county)	100%	real	county	-

### 2.1 Target variables

We obtained the number of **COVID-19 confirmed cases** and **deaths** from the *USAFacts* website [3], which is sourced from the *US Centers For Disease Control and Prevention (CDC)* [4]. This data source records the cumulative number of confirmed COVID-19 cases and the cumulative number of confirmed COVID-19 deaths for each county and day from the first case report on January 22, 2020. We obtained the new cases (and deaths) per day by subtracting the total of each day from the previous. This resulted in negative values for some counties on some days. After contacting the data source, we learned that the government agencies update their reported accumulative numbers to be lower than the previous day if they obtain a more accurate count by re-examining their medical records. Thus, the negative values generated in the daily confirmed cases and deaths data are related to the additional counts that were incorrectly reported. In the processed dataset, we used the following method to solve this problem. We set the entry of each day with a negative value to zero and add this negative value to the previous day's entry. Then if the result of the sum is negative, we repeat this step until one day's entry sums up to a positive value. So far, the repetition was never required as the resulting sums were always non-negative.

### 2.2 Fixed features

#### 2.2.1 Demographic features

We obtained most of the demographic data – viz., the **total population**, age and sex distribution, number of housing units, and county **area** – from the US Census Bureau websites; here we report 2018 values [5,6]. Sex distribution is included in the data with the variable named **proportion female**, which contains the ratio of female population to the total population of each county and the age distribution corresponds to the 18 variables (i.e. **age 0_4, age 5_9, age 10_14, age 15_19, age 20_24, age 25_29, age 30_34, age 35_39, age 40_44, age 45_49, age 50_54, age 55_59, age 60_64, age 65_69, age 70_74, age 75_79, age 80_84**, and **age 85 or higher**), each specifying the percentage of residents in the specific age group in the county.

To calculate the **academic population ratio**, we first collected the total enrollment of university and college students and academic staff that each county reported in the fall of 2018 using data from the *National Center of Education Statistics (NCES)* [7]. This was divided by the population of that county in 2018 to calculate the ratio.

Another factor that might affect COVID-19 preventive policy adherence is the education level distribution of each county. This factor may also approximate the portion of people who work remotely. The related variables in the data are a percentage of educated people at the three education levels ‘**less than high school diploma**’, ‘**high school diploma**’, and ‘**some college or associate's degree**’, which we downloaded from the *United States Department of Agriculture (USDA)* [8].

Immigrant students are those who enrolled in the fall of 2018 in any institution in the county but reside in another state [7]. We derived the **immigrant student ratio** by dividing the total number of immigrant students by the total county population. This factor could provide an estimate of a possibly higher rate of virus transmission in areas such as college towns.

**Religious congregation ratio** is calculated by dividing the total number of active members of a county's religious congregations reported in 2010 by the total county population [9].

We calculated the overall death ratio for each county regardless of the cause of the death based on the number of deaths per 100,000 residents in 2018, which is collected from CDC [10].

#### 2.2.2 Health facilities and risk factors features

The information about a county's health facilities is included in the data mostly on a per capita basis. We collected the number of **intensive care unit (ICU) beds** and **ventilator capacity** data from the *Tableau Public* website [11] and the total number of **hospital beds** per 1000 individuals from the *Urban Institute* website [12], which is sourced from the *American Hospital Association Annual Survey* Database [13]. Then we derived the per capita information about these facilities using each county's population. **Percent of smokers** and **Percent of diabetes** show the percentage of adult smokers and diabetic adults in the total population of the county, respectively. Our data source for smoker and diabetes ratios and **percent of insured residents** was the *County Health Rankings and Roadmaps* website [14].

#### 2.2.3 Geographic features

*Airport distance* for each county shows the distance to the nearest international airport, obtained by considering the “great circle distance” calculated through the latitude and longitude of the airport and the county center. Only airports with more than ten daily passengers, on average, are considered (using data prior to COVID-19) and the airport distance for the counties with one or more airports inside them is set to zero. This feature is included in the data to reflect the vulnerability of the county to the possible infections caused by arrival flights from the countries affected by the virus.

The passenger load for each county is the passenger load of the nearest international airport to that county, and if a county includes more than one international airport, the passenger load equals the total passenger load of these airports. These data were collected from the *United States Department of Transportation* and *OpenFlights*  websites [15,16]. We derived the **passenger load ratio** by dividing the passenger load by the total population of the county.

The **number of meat plants** shows the number of meat and poultry processing plants in each county, collected from the *United States Department of Agriculture* website [17]. Meat and poultry plants are reported as high-risk places for COVID-19 virus transmission [18].

#### 2.2.4 Economic and other features

Our economic features for each county, including the **median household income** and **GDP per capita**, are both based on data reported in 2018, obtained from the *Census Bureau* and *the United States Bureau of Economic Analysis* websites [19,20]. The governing **political party** data was collected from *Wikipedia* [21] and is included to investigate the possible impact of politics and political views on the adopted preventive policies, adherence encouragement, and the number of cases and deaths reporting system.

We also added the **population ratio** of each county in the state to the data, which can be used to derive county scaled features from features with a state scaled basis.

### 2.3 Temporal features

#### 2.3.1 Climate features

Our source for climate data, the *Daily Summaries* dataset [22], contains daily **precipitation** and daily maximum, minimum, and average values for **temperature** each day. **Precipitation** is considered as a relative measure of humidity that along with **temperature** can determine the climatic characteristic of a region, which is known as an influencing factor in the COVID-19 pandemic [23,24].

#### 2.3.2 Social distancing features

Our social distancing data source, *Unacast* [25], is based on mobile location data. In collecting this data, users consent to opt-in and can opt-out by filling out a form on the data source website. This data source contains four different metrics: **social distancing travel distance grade, social distancing visitation grade, social distancing encounters grade**, and an overall average score of these three metrics named **social distancing total grade**. Each of these grades determines the percentage of reduction in a measure of unnecessary social activities (e.g. traveling, human encounters, non-essential visitation) compared to the pre-COVID-19 period, which is translated into letter grades, as described in Table 1.

The **encounters grade** is based on the proximity of two devices within a circle of radius 50m for less than an hour, counted as one encounter. This grade shows the decrease in human encounter density (number of encounters in the county per square km of land area) compared to the baseline, where the baseline is defined as the national average encounter density during the four weeks before the COVID-19 outbreak (February 10th - March 8th). The reason why the baseline is defined over the whole nation is to assign lower grades to denser areas, even if they witness fewer encounters compared to the pre-COVID-19 period. Namely, dense areas have a high infection risk, even if they were denser in the past.

The **visitation grade** indicates the percent change in visits to non-essential venues compared to the pre-COVID-19 period. Non-essential venues include but are not limited to restaurants (multiple kinds), department and clothing stores, jewelers, consumer electronics stores, cinemas and theaters, office supply stores, spas and hair salons, gyms and fitness/recreation facilities, car dealerships, hotels, craft, toy and hobby shops. The average visitation for each day of the week prior to the COVID-19 outbreak (between February 10th to March 8th) is considered as the baseline. The percent change is calculated by comparing those baselines to visits on the corresponding days of the week post-outbreak (March 9th onwards).

The **travel distance grade** simply shows the percentage reduction in average distance traveled in each county for each day. The highest grade for this metric represents more than 70% reduction in average distance traveled. This threshold is selected based on the experience gained from Italy because they implemented some of the most strict social distancing policies, which resulted in a 70% to 80% reduction. Therefore, Unacast expects a maximum of 70% reduction in distance traveled under a total shot-down.

We also used the *Google Mobility Reports* data source [26] to include additional social distancing adherence features. This data is collected from the information of users who have opted-in to location history for their google account and consists of 6 variables (i.e. **retail and recreation mobility percent change, grocery and pharmacy mobility percent change, parks mobility percent change, transit stations mobility percent change, workplaces mobility percent change, residential mobility percent change**), each representing the change in visits and length of stay in a specific place category compared to the baseline. The baseline for each place category and each date is determined based on the day of the week, and its value is the median number of visitors to that place on that day of the week in the five-week pre-COVID-19 period from January 3 to February 6, 2020. Place categories include **parks, transit stations, residences, workplaces, grocery stores and pharmacies, retail shops and recreation centers**. Category of **parks** consists of places such as local and national parks, public beaches, marinas, dog parks, plazas, and public gardens. The **transit station** category represents all the public transport hubs like taxi stands, bus, train, and subway stations. **Residential** and **workplaces** refer to places of residences and places of work in each county. **Grocery stores and pharmacy** categories include different kinds of food shops and drug stores. And places such as restaurants, shopping centers, museums, and libraries belong to the category of **retail shops and recreation centers** [26].

#### 2.3.3 Other features

**Daily state tests** refer to the number of daily tests performed in each state. These numbers were obtained using statistics from multiple type COVID-19 tests including antibody, antigen, and PCR. This data was downloaded from the *COVID Tracking Project* (https://covidtracking.com/).

The **weekly admission, weekly reported total inpatient beds, weekly occupied inpatient beds, weekly reported total ICU beds**, and **weekly occupied ICU beds** are the reported data on health facility utilization from the county hospitals, collected from *US Department of Health and Human Services* [27]. The data is recorded weekly, where a week is defined as the 7 day period from Friday to Thursday. The **weekly admission** is the weekly average of the total number of patients, adult or pediatric, confirmed with COVID-19 at the time of admission, who are admitted to an inpatient bed in the hospitals of that county. The **weekly reported total inpatient beds** is the weekly average number of the total number of staffed inpatient beds including all overflow, observation, and active surge/expansion beds used for inpatients (including all ICU beds) reported from all of the hospitals of a county during the week. Similarly, the **weekly reported total ICU beds** is the weekly average number of total staffed inpatient ICU beds. The **weekly occupied inpatient beds** and **weekly occupied ICU beds** is the weekly average number of total staffed inpatient beds and ICU beds respectively, occupied in all hospitals of a county during the week [27].

Since the data source is at the hospital level, we first obtained the county level data for each feature by aggregating the weekly sum of the total number of reported cases (e.g., weekly sum of the total admissions and occupied ICU beds) over the hospitals in each county. Then we derived the weekly average total number of cases by dividing this value by seven. To determine the hospitals that belong to each county, if the county code of the hospital was not recorded in the data, we used the zip code to determine the county of that hospital. If the zip code of a hospital was shared among several counties, we distributed the reported cases among the counties proportionally to their populations. This ensures that the sum of the number of reported cases over the counties is the same as the reported cases at the national level.

The **percent of vaccinated residents** represents the percentage of the residents of each county who are fully vaccinated – i.e., who have had both doses of a two-dose vaccine or one dose of a single-dose vaccine [28]. The vaccination data was downloaded from CDC [28].

The **virus pressure** at county $x_{i},\;$and day t, denoted by $V(x_{i},t)$, is defined based on the number of COVID-19 cases in the neighboring counties:

(1) $$V\left(x_{i},t\right)=\frac{\sum_{x_{k}\in N\left(x_{i}\right)}C\left(x_{k},t\right)}{\left|N\left(x_{i}\right)\right|}$$

where $C(x_{k},t)$ denotes the number of COVID-19 cases in county $x_{k}$ at day *t*, and $N(x_{i})$ is the set of all adjacent counties that share a border with county $x_{i}$, excluding $x_{i}$ itself. To verify the importance of this feature, we used the *mRMR (minimum Redundancy Maximum Relevance)* [29] method to rank the features based on the target variable: the number of COVID-19 confirmed cases in each county. This method iteratively selects features with high correlation with the target variable but low correlation with those features already selected higher in the ranking. **Virus pressure** was ranked 3rd, representing its impact on this target variable.

### 2.4 Data processing

Table 1 shows the percentage of missing values for each feature. To obtain the processed dataset, we removed those counties that were missing any of the fixed features. For each of the temporal features, if a county was missing its values for only some of the recorded dates, we imputed these missing values as explained below; however, if there were missing values over all of the recorded dates, we removed the county from the dataset. This resulted in the elimination of a total of 1181 counties.

We imputed the missing values of each feature for the remaining counties. In general, we used the *KNN imputer* [30] to impute the missing values of a feature based on the other non-missing values of that feature, for that county.  We discuss the few exceptions, below.

To deal with the daily average **temperature** (which is missing 79% of its values): If the corresponding minimum and maximum daily temperatures for that county were reported, we would impute the mean as the average of those two values. We used the KNN imputer (see above) to impute the remaining 41% of the missing values for the instances that did not include the minimum and maximum temperatures.

For the social distancing features, our data source started recording the data only beginning February 24, 2020. We set the values for the previous days (January 22 to February 23) to the lowest grade for each social distancing feature, for each county. That is, we assumed no social distancing policies were imposed prior to that time. Note the **encounters, travel distance**, and **total grade**, each still had 1% missing values for the remaining dates (post 24 February) and the **visitation grade** had 18% missing values – here, we use the KNN imputation system described above.

The Google mobility data started recording from February 15, 2020. We imputed the mobility features for the previous days (January 22 to February 14) in the same way as social distancing features. That is, we assumed the lowest percent change (zero), representing no change in mobility trend compared to the pre-COVID-19 period. The percent of change in mobility trends for the rest of the dates in **parks, transit stations, residences, workplaces, grocery stores and pharmacies, retail shops and recreation centers** had 82%, 72%, 58%, 26%, 56%, and 51% missing values respectively. Here, we again used the KNN imputer.

The number of **daily state test** data contained negative values for some states and days, and the first recording date varied from state to state. We considered the negative numbers as missing values and imputed them along with the unrecorded feature values.

### 2.5 Data records

The version of the dataset at the time of submission, containing data from January 22, 2020 to June 10, 2021, has been archived in figshare [31], and the latest version of the dataset is publicly available in our Github repository https://github.com/network-and-Data-Science-IUT/covid-19. We included 2 datasets: (i) the raw dataset (“raw_data.csv”) with the negative and missing values, and (ii) the processed dataset (“imputed_data.csv”) where the counties with missing values are all imputed or removed from the dataset. Each row in the datasets corresponds to a specific county and date. Counties and their associated states are represented using their name and zip code [32]. Table 1 specifies the name, type, spatial scale, and description of each feature and also the percentage of their values  available in the raw dataset. Note that being a derived feature, **virus pressure** exists only in the processed dataset. Moreover, we removed the features **parks, transit stations**, and **residences mobility percentage change** from the processed dataset as they had no recorded value for a large number of counties. On the other hand, since the data source for features **weekly admission, weekly reported total inpatient beds, weekly occupied inpatient beds, weekly reported total ICU beds**, and **weekly occupied ICU beds** starts recording the data from July 31, 2020, data on these features does not cover the whole range of recorded dates in our data and hence we removed these features from processed data. The size of the current raw and processed datasets are 688 and 443 MB.  We plan to update the datasets until the end of 2021.

**Fig. 1:**
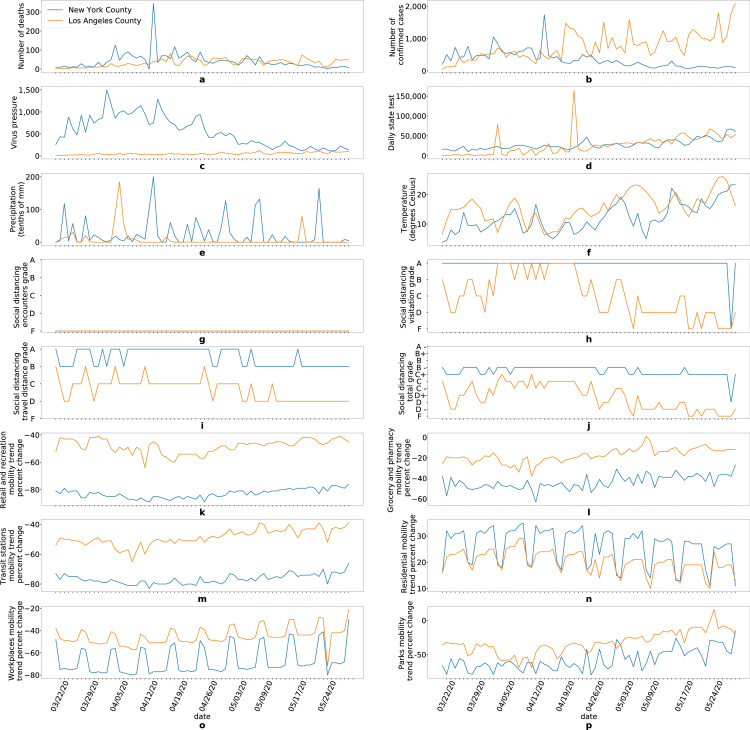
Evolution of the daily based temporal features (a) number of deaths, (b) number of confirmed cases, (c) virus pressure, (d) daily state test, (e) precipitation, (f) temperature, (g) social distancing encounters grade, (h) social distancing visitation grade, (i) social distancing travel distance grade, (j) social distancing total grade, (k) retail and recreation mobility percent change, (l) grocery and pharmacy mobility percent change, (m) transit stations mobility percent change, (n) residential mobility percent change, (o) workplaces mobility percent change, (p) parks mobility percent change, during the peak days of the COVID-19 outbreak.

For illustration, Fig. 1 indicates almost all daily temporal features for the New York and Los Angeles Counties over the peak days of the disease outbreak (March 22, 2020, to May 30, 2020). As seen, the **social distancing encounters grade** has not changed over this time. This is probably because both New York and Los Angeles counties are densely populated areas, even during the pandemic, meaning the preventive policies do not receive a grade better than 'F' for this measure.

### 2.6 Technical validation

We verified the compatibility of the number of **COVID-19 confirmed cases** and **deaths** with the Worldometer Website [33] reports by randomly choosing 15 counties then manually comparing the number of confirmed and deaths in our data with the Worldometer data; we found no inconsistencies. We also checked each counting variable for negative values using the *pandas package* [34]. This identified negative values for the number of newly confirmed COVID-19 cases and deaths, which appeared to be part of the reporting procedure: if the reporters realize an over-reporting in yesterday's number of cases or deaths, they reduce it from today's number, which can result in a negative value. We imputed these negative values using an appropriate method described in the Methods section. When adding a feature to the dataset, we used the pandas package to identify and remove duplicate feature records for the same county and date. In addition, by looking at the summary of each feature, including its min, max, and mean values, as well as randomly observing some of the values of that feature, we checked if the values belong to the logical range of that feature. For example, the raw data collected for temperature included values in the range [−500,500] for Celsius. After contacting the corresponding website [22], it appeared that we had to divide by 10 to obtain the correct values in Celsius. None of the other features had this issue.

### 2.7 Code availability

Data collection and preparation were done using the python programming language. We used the *json* and *requests* packages [35,^36^]to collect data and the *scikit-learn* package [37] to impute missing values. To obtain climate data from the data source *Application Programming Interface (API)* [22], we needed the weather stations' information. We used the data source API [38] to obtain weather station information for all counties on May 14, 2020. Since then, we used that information to obtain climate feature data on a daily basis from the specified API [22]. We also collected social distancing data using the data source API [25], but we downloaded the rest of our dataset features manually or automatically using direct links to the data sources. The codes used to collect and prepare the datasets are available in our Github repository [39].

## Ethics Statement

The paper is not currently being considered for publication elsewhere.

## CRediT authorship contribution statement

**Arezoo Haratian:** Data curation, Writing – original draft. **Hadi Fazelinia:** Data curation, Resources, Writing – review & editing. **Zeinab Maleki:** Data curation, Supervision, Writing – review & editing. **Pouria Ramazi:** Data curation, Supervision, Writing – review & editing. **Hao Wang:** Supervision, Writing – review & editing. **Mark A. Lewis:** Supervision, Writing – review & editing. **Russell Greiner:** Supervision, Writing – review & editing. **David Wishart:** Supervision, Writing – review & editing.

## Declaration of Competing Interest

The authors declare that they have no known competing financial interests or personal relationships which have, or could be perceived to have, influenced the work reported in this article.

## Data Availability

USA covid-19 data (Original data) (figshare).
